# *In Vitro* Analysis of Pyrogenicity and Cytotoxicity Profiles of Flex Sensors to be Used to Sense Human Joint Postures

**DOI:** 10.3390/s140711672

**Published:** 2014-07-01

**Authors:** Giovanni Saggio, Luigi Bianchi, Silvia Castelli, Marilina B. Santucci, Maurizio Fraziano, Alessandro Desideri

**Affiliations:** 1 Department of Electronic Engineering, University of Tor Vergata, via del Politecnico, 1-00133 Rome, Italy; 2 Department of Civil Engineering and Computer Science, University of Tor Vergata, via del Politecnico, 1-00133 Rome, Italy; E-Mail: luigi.bianchi@uniroma2.it; 3 Department of Biology, University of Tor Vergata, Via della Ricerca Scientifica, 1-00173 Rome, Italy; E-Mails: cstslv00@uniroma2.it (S.C.); santucci@scienze.uniroma2.it (M.B.S.); Fraziano@bio.uniroma2.it (M.F.); desideri@uniroma2.it (A.D.)

**Keywords:** flex sensors, pyrogenic potential, cytotoxicity, biocompatibility, human joints

## Abstract

Flex sensors can be usefully adopted as mechanical-electrical transducers to measure human joint movements, since their electrical resistance varies proportionally to the angle assumed by the joint under measure. Over time, these sensors have been investigated in terms of mechanical and electrical behavior, but no reports have detailed the possibility of their adoption not just on top but under the human skin of the joint. To this aim, our work investigated *in vitro* the pyrogenic potential and cytotoxicity of some commercially available flex sensors as a first step toward the necessary requirements regarding their biocompatibility, to predict possible foreign body reactions when used *in vivo*. Results demonstrated that some specific flex sensors satisfy such requirements.

## 1. Introduction

Flex sensors are widely used to sense the static and dynamic postures of human joints, transducing angles into electrical resistance values. The reason for their usage relies on their good reliability and the repeatability of the measurements [1], in spite of their lightness and cost effectiveness. Their main application has been the sensing of flex/extension and abdu/adduction movements of the hand's fingers [2]. However, they have been also used to measure rotations of other human joints, such as the elbow [3] and ankle [4], to reveal the kinematics of the trunk [5] and shoulder [6], or to furnish an aid in gait analysis [7] and orthotic appliances [8]. This is because these sensors can be used to sense bending angles both with fixed and/or changing curvature radius too [9].

Flex sensors have been investigated in terms of their mechanical properties [10], of their electrical quasi-static [11] or fast [12] readouts, and of their electrical response related to their shape [13]. However, as far as we know, there have been no efforts to evaluate their pyrogenic and cytotoxic potential, which we aim to investigate here, to reveal potentially adverse effects, if any.

In this context, a classical adverse effect that occurs, following implantation of biomaterials within the body, is a sterile acute inflammatory response called foreign body reaction. Inflammasome activation and IL-1β release represent the early molecular events leading to the initial recruitment of neutrophils and subsequently of macrophages, which over a period of weeks to months, mediate the next recruitment of fibroblasts releasing collagen, whose final outcome is the encapsulation and isolation of the foreign body [14–17].

Here, we intend to establish the cytotoxic and pyrogenic response of these kinds of sensors as a first step in considering the possibility to use them directly under the human skin, which can be relevant in special applications or events. We refer, for instance, to the possibility of semi-permanent insertion under the skin of the flex sensors for people who experienced severe injuries, with reduction of some motor activities and/or joints' degrees of freedom (DOFs). The semi-permanent insertion can furnish a particular solution in just some special cases, of course, but can sometimes assure a better quality of life, avoiding the permanent usage of external supports embracing the sensors. Under-skin sensor(s) can provide a continuous stream of data, useful to monitor the reduced motor activities, and/or to furnish information about the effectiveness of orthoses, and/or to drive electro-mechanical prosthesis or external motor actuators.

In such a framework, this work intends to evaluate *in vitro* the cytotoxicity and the pyrogenic potential of some of the most widely used flex sensors. This is also because, as far as we know, no commercially available sensors, capable to measure joint's flexion, exist specifically designed for epicutaneous use.

## 2. Materials and Methods

In this section, we treat of the nature of the used sensors, their electrical behavior, and the way they were handled to determine their pyrogenic potential and cytotoxicity.

### 2.1. Sensors

Flex sensors are commonly made of brittle ink carbon resistive elements printed on top or within a thin flexible plastic substrate, shaped as a stripe. Micro gaps result within the ink proportionally with flex angles, so that higher flexion produces higher electrical resistance. Two metal terminals realize conductive connections.

**Figure 1.**
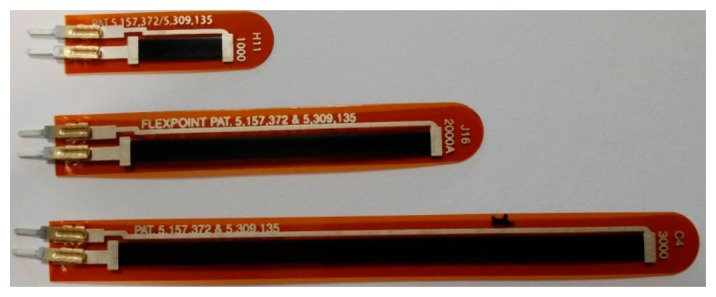
2.54 cm (1”), 5.08 cm (2”), and 7.62 cm (3”) long flex sensors by Flexpoint Inc. The darker regions are covered with the carbon based resistive elements. At the left ends the metallic terminals for electric connections.

Currently, three main brands of flex sensors are affordable and easily available: Spectra Symbol (Salt Lake City, UT, USA), Abrams-Gentile (New York, NY, USA), and Flexpoint Inc. (South Draper, UT, USA). The latter produces flex sensors with the greatest resistance variation and higher measurement repeatability and accuracy, within few degrees [18], when bent; this was the reason to select them for our study. Dimensions of the sensor's strip are approximately 7.1 mm wide, 0.1 mm thin with commercial lengths of 2.54 cm, 5.08 cm and 7.62 cm (Figure 1). In particular, we investigated the 5.08 cm version in three variants: non-encapsulated, polyester-encapsulated and polymide-encapsulated.

**Figure 2.**
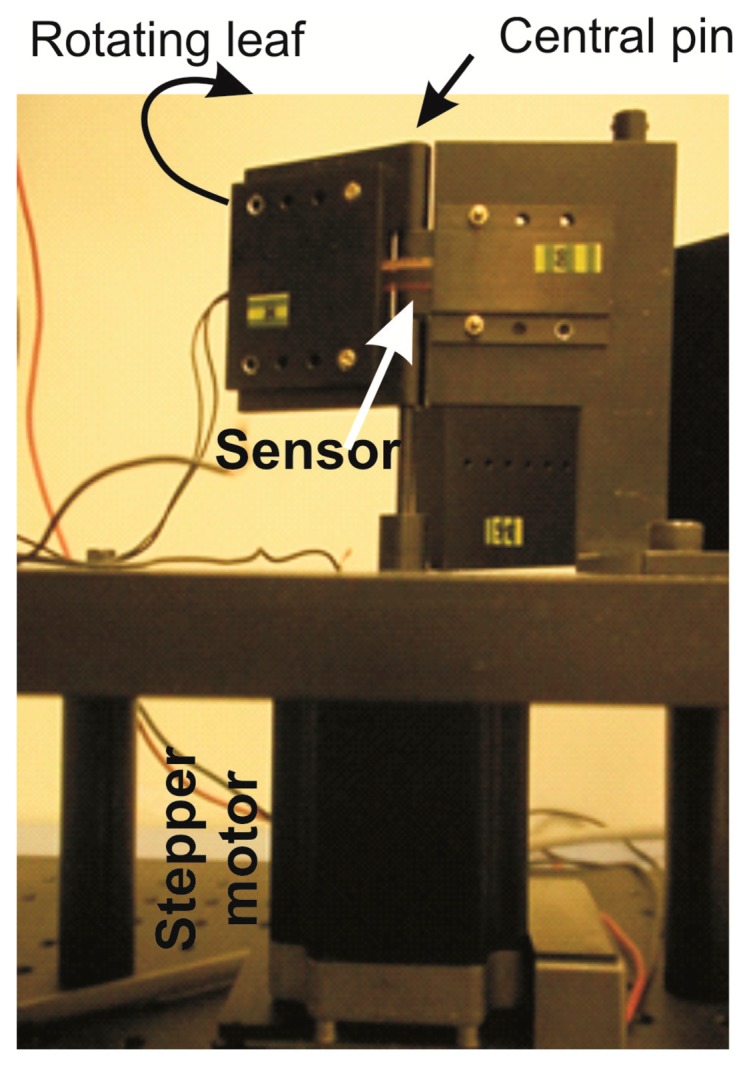
The setup was to measure the electrical behavior of the sensor when flexed to different angles. A stepper motor moves the rotating leaf of a hinge and a multimeter records the resistance value of the sensor. A Labview^®^ routine controls the overall system.

In order to determine their electrical behavior, so as to associate an electrical resistance value to any bending angle, we performed quasi-static measurements using a homemade semi-automatic measurement set-up (Figure 2). It consisted of a hinge with a central pin around which the sensor was automatically flexed by a stepper motor (PD-109-57 by Trinamic, Hamburg, Germany), controlled by a Labview^®^ routine (details reported in [11]). One leaf of the hinge was rotated forwards and backwards from 0 to 90 degrees, stepped 5 degrees, and at each step the new resistance value of the sensor was acquired by a digital multimeter (34405A by Agilent, Santa Clara, CA, USA). No further investigation was necessary, since any other aspects, in particular the accuracy, the reliability [19], and the stability over time [18], were already investigated. Ten measurement cycles were performed on sensors both before and after the pyrogenic and biocompatibility tests, so to compare the respective results in order to evaluate possible changes in their resistance variation *vs.* bending angle, if any.

### 2.2. Sensor's Handling for Biological Assays

We preliminarily washed the sensors with 70% ethanol and water, then dried and sterilized them under UV light for 30 min, and finally used them for the cell viability and pyrogenic tests. The carbon/plastic part and, in some specific cases the metal part too (details in the following sections), were incubated in Dulbecco's Modified Eagle Medium (DMEM) for three days at 37 °C, to permit the release of the soluble components in the medium, and study their potential toxicity. Foetal bovine serum and L-glutamine can degrade if incubated at 37 °C over an extended period, for this reason we preferred to add these two components to the culture medium immediately before the experiment.

### 2.3. Cell Cultures

Human keratinocytes (HACAT) were grown in DMEM medium supplemented with 10% heat-inactivated fetal bovine serum, penicillin (100 units/mL), streptomycin (100 units/mL), and 4 mmol/L glutamine in a humidified atmosphere of 95% air and 5% CO_2_ at 37 °C.

### 2.4. Cell Viability Assay

Cell proliferation was evaluated by using the CellTiter 96 Aqueous One Solution Cell Proliferation Assay (Promega, Madison, WI, USA). The assay used the tetrazolium compound 3-(4,5-dimethylthiazol-2-yl)-5-(3carboxymethoxyphenyl)2-(4-sulphenyl)-2*H*-tetrazolium, inner salt (MTT). In particular, 104 cells were seeded in 96 well plates in the presence of a medium previously incubated with the only plastic part of polyester, polyimide and no-encapsulated sensors, and grown for 24 h. Later, the cell proliferation was monitored adding 20 μL/well of MTT for 2 h at 37 °C in a humidified, 5% CO_2_ atmosphere. The absorbance at 490 nm was recorded using an ELISA plate reader [20]. The final value represented the mean ± SD of three replicates. As a positive control of the assay, we used cells grown in fresh DMEM medium.

### 2.5. Pyrogenic Potential in Whole Blood Assay

The release of IL-1β was used to test the biocompatibility of the sensors since their release is known to be directly associated with the biocompatibility of medical devices [21].

Briefly, blood was drawn from three healthy volunteers (2 males, 1 female) into heparinised tubes. Polyester, polyimide and non-encapsulated sensors were incubated in 24 wells plates, with 100 μL of fresh blood diluted in PBS, in a total volume of 1 mL for 24 h at 37 °C and 5% CO_2_. As positive control, blood was stimulated with 100ng/mL Lipopolysaccaride (LPS), whereas as negative control the blood was not stimulated. Cell-free supernatants were collected and stored at −80 °C until use. The release of IL-1β in the supenatant was detected by ELISA by a commercially available kit (Thermo Scientific), and used according to the manufacturer's instructions.

## 3. Results

### 3.1. Cytotoxicity

**Figure 3.**
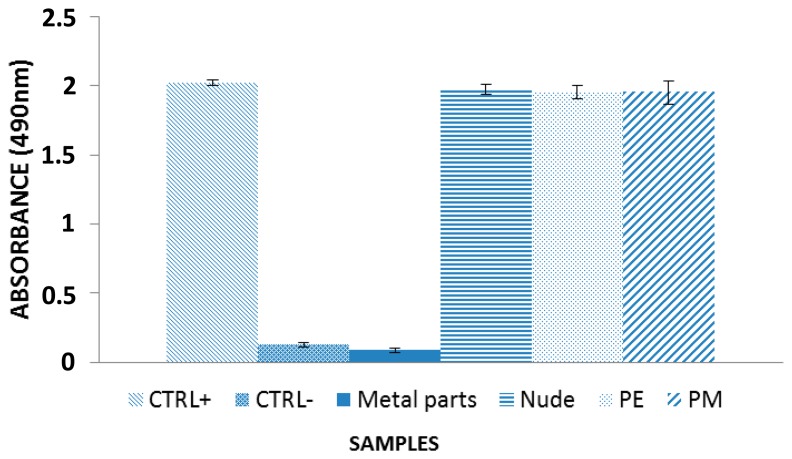
Cells viability evaluated with MTT assay. “Nude”, “PE” and “PM” stand respectively for “non-encapsulated”, “polyester-encapsulated” and “polymide-encapsulated” sensors. HACAT cells were incubated overnight in medium pre-incubated with: the metal parts, no-encapsulated (“nude”) sensor, polyester (“PE”) and polyimide (“PM”) encapsulated sensors. CTRL-; negative control, cells incubated with 1% triton-X100. CTRL+; positive control; cells grown in fresh DMEM. Results are indicated as means ± SD of three different measures.

Cell proliferation assays were carried out to estimate the cytotoxicity of the different regions/materials of the sensor. To this aim, the different sensors were incubated in DMEM for three days, as above described, and we used the MTT (3-(4,5-dimethylthiazol-2-yl)-5-(3-carboxymethoxyphenyl)- 2-(4-sulfophenyl)-2*H*-tetrazolium) assay for cytotoxicity. This is based on the cleavage of the yellow tetrazolium salt MTT to purple formazan crystals in metabolically active cells, the colorimetric signal being proportional to the number of viable cells. The results, shown in Figure 3, indicate that the encapsulated or non-encapsulated sensors do not show any sign of cytotoxicity, and can be subsequently considered to evaluate their possible pyrogenic effect. On the other hand, the sensors demonstrated high cytotoxicity when their metal parts were incubated with the medium used in the assay, just because of the interaction between the cells and the metallic parts only.

### 3.2. Analysis of Pyrogenic Potential of the Different Flex Sensors

**Figure 4.**
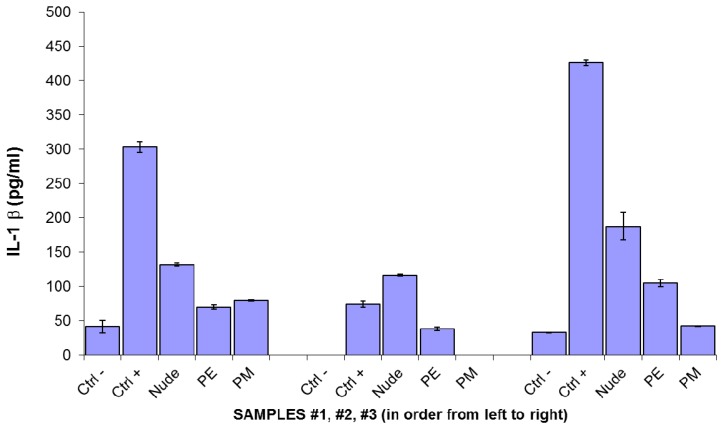
IL-1β release in a whole blood assay. Whole blood obtained by three different healthy donors was collected and incubated overnight in absence (−ve controls) or in the presence of non-encapsulated (“nude”), polyester-encapsulated (“PE”) or polyimide-encapsulated (“PM”) sensors. +ve controls consisted of LPS. Results are expressed ±SD of three different culture determinations.

The pyrogenic potential of any biomaterial is an important component of biocompatibility analysis, with mounting evidence showing that IL-1β production by host innate immunity can induce foreign body reaction and result in implant failure [21]. In this context, foreign body reaction was assessed *in vitro* by evaluating IL-1β production by whole blood samples exposed *in vitro* to the different flex sensors (the blood came from three healthy volunteers). Human whole blood was brought into contact with the non-encapsulated, polyester or polyimide encapsulated flex sensors and the release of cytokine IL-1β used as an *in vitro* assay predictive of foreign body reaction, was measured by ELISA assay. The results shown in Figure 4 demonstrate a significant IL-1β release both in non-encapsulated or polyester encapsulated sensors, although significantly lower than that observed in LPS stimulated positive control. The polyester encapsulated sensor showed a lower proinflammatory profile. However, in all tested blood samples, the polyimide-encapsulated flex sensors display an IL-1β release comparable to that of the negative control and allow us to consider it for further pre-clinical studies.

### 3.3. Resistance Measurements

**Figure 5.**
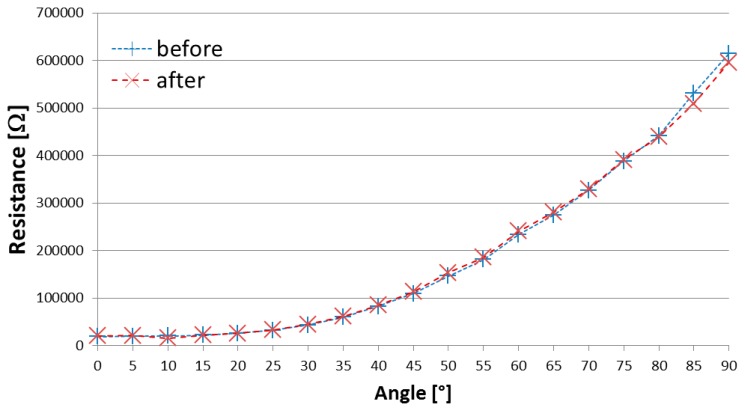
Two resistance *vs.* bending angle characteristics of one sample of polymide-encapsulated 2” flex sensor. The two curves were recorded before and after the tests. There is no evidence of any degradation of the electrical behavior of the sensor. This plot is for just one sample, but the same kind of behavior was recorded for all the sensors evaluated.

Before and after the *in vitro* pyrogenicity and cytotoxicity tests, we measured the electrical behavior of the sensors, in terms of resistance *vs.* flex angle pairs, to demonstrate potential degradation of sensor's response following cell culture, if any. Figure 5 shows the electrical behavior of one sample of polymide encapsulated flex sensor, and it is relevant to observe that there is no hysteresis effect and no significant degradation. The same occurred for all the sensors tested.

## 4. Discussion and Conclusions

Biomaterials are widely used in regenerative medicine. Worldwide there are over 100 different commercially available biomaterial-based medical devices, with different composition and formulation for clinical use [22]. Although these materials are commonly used and considered relatively biocompatible, a number of studies have shown that they may have biocompatibility issues [23]. In this study, we focused our attention on the pyrogenicity and cytotoxicity profiles of particular devices, such as flex sensors, which are capable of converting mechanical bending effects into an electrical value. In particular, we analyzed the most adopted commercially available ones, never considered before for epicutaneous use. The implantation of such flex sensors into experimental animals is the most effective experiment to define their full biocompatibility, however this work represents a first step to test their capability to induce a direct cytotoxic and/or pyrogenic effect. Permanent cell lines are generally employed for *in vitro* cytotoxicity, which can be monitored by a MTT assay, according to the international standard ISO 10993-5. Given that the flex sensors described herein should ideally be implanted under the skin, we used a model of keratinocyte cell line in a short-term medium extraction test, where DMEM was incubated with the different sensors for 24 h, and then used to culture HACAT cell line for cytotoxic evaluation after 3 days by MTT assay. Reported results show a complete absence of cytotoxicity in the cell model used. Another parameter to evaluate biocompatibility issues is given by the prediction of foreign body reaction. This reaction is mediated by immune cells, such as leukocytes and platelets, which intervene in order to protect the body from the foreign object [24], and is caused by the tissue injury resulting from implantation of the device, as well as the exposure of danger associated molecular pattern on the surface of the device in the body [25]. The main stages of this process include acute inflammation, chronic inflammation, the formation of granulomatous tissue and the walling off the device by a vascular, collagenous fibrous capsule [26]. This fibrous wall confines the implanted device and prevents it from interacting with surrounding tissues thus leading to failure implantation. One of the earlier soluble mediators of foreign body reaction is IL-1β, which is immediately released following tissue injury and/or the recognition of danger associated molecular pattern [27] on the surface of the device, and can be used as a predictable marker of inflammatory response [28].

In this study, we used whole blood based assay in order to get information about *in vitro* reactogenicity of some of the most common flex sensors, by different immune cell types. Because the assay mimics the natural environment, whole blood culture may be the best environment to study cell activation and cytokine production *in vitro*. In our work, the whole blood based assay was able to discriminate the levels of response induced by three different types of flex sensors, which did not show any sign of cytotoxicity but identified polyimide-encapsulated sensor only as the less reactogenic one, so to become a possible candidate for future experiments of implantation in small animal models. Reasonably, we do not expect a different answer even for longer incubation time (even if further long-term tests will be performed) and so implantation will be the next programmed experiment to verify a full *in vivo* biocompatibility of the sensor.

In conclusion, the present study reports evidence concerning the *in vitro* pyrogenic potential and cytotoxicity of some commercially available flex sensors, in order to assess their biocompatibility, and in view of a possible semi-permanent insertion under the skin for people who have experienced severe injuries to continuously monitor their residual motor activities. The study demonstrated that, among the adopted three types of flex sensors, the polyimide-encapsulated one showed the lowest pyrogenic effect, with no relevant cytotoxicity, and displayed a low proinflammatory profile in the discussed experimental models.

Finally, the impact on sensor performances, in terms of changes in the resistance *vs.* bending angle characteristics, was practically negligible, since no electrical mismatches resulted between resistance values measured before and after all the tests. In a spirit of completeness, the authors plan to perform tests of accuracy, reliability and stability over time to determine differences after the treatments, if any. The overall results suggest such flex sensors for consideration in future pre-clinical studies.
